# Coherent backscattering in quasi-ballistic ultra-high mobility GaAs/AlGaAs 2DES

**DOI:** 10.1038/s41598-018-28359-0

**Published:** 2018-07-03

**Authors:** R. L. Samaraweera, H.-C. Liu, B. Gunawardana, A. Kriisa, C. Reichl, W. Wegscheider, R. G. Mani

**Affiliations:** 1 Department of Physics and Astronomy, Georgia State University, Atlanta, 30303 Georgia; 2 Laboratorium für Festkörperphysik, ETH Zürich, CH-8093 Zürich, Switzerland

## Abstract

A small and narrow negative-magnetoresistance (MR) effect that appears about null magnetic field over the interval −0.025 ≤ *B* ≤ 0.025 *T* in magnetotransport studies of the GaAs/AlGaAs 2D system with *μ* ≈ 10^7^*cm*^2^/*Vs* is experimentally examined as a function of the sample temperature, *T*. The temperature dependent magnetoresistance data were fit using the Hikami *et al*. theory, without including the spin-orbit correction, to extract the inelastic length, *l*_*i*_, which decreases rapidly with increasing temperature. It turns out that *l*_*i*_ < *l*_*e*_, where *l*_*e*_ is the elastic length, for all *T*. Thus, we measured the single particle lifetime, *τ*_*s*_, and the single particle mean free path *l*_*s*_ = *v*_*F*_*τ*_*s*_. A comparison between *l*_*i*_ and *l*_*s*_ indicates that *l*_*i*_ > *l*_*s*_. The results suggest that the observed small and narrow magnetoresistance effect about null magnetic field could be a manifestation of coherent backscattering due to small angle scattering from remote ionized donors in the high mobility GaAs/AlGaAs 2DES.

## Introduction

Advancements in the molecular beam epitaxy growth of the GaAs/AlGaAs two dimensional electron system (2DES) now routinely yields high-quality heterostructures with 2D electron mobilities *μ* ≥ 10^7^ *cm*^2^/*Vs*, making this the most disorder free 2D material system in existence. In such specimens, the transport or elastic length, ł_*e*_ = *v*_*F*_(m*μ/*e*) = *v*_*F*_*τ*_*e*_, where *v*_*F*_ is the Fermi velocity, m^*^ is the effective mass, *e* is the charge, and *τ*_*e*_ is the transport/elastic time, can be comparable to the sample size even in mm-scale specimens, implying quasi ballistic transport in the absence of a magnetic field. Such high quality material has demonstrated new 2D physical phenomena such as the photo-excited zero-resistance states^1,2^ and 1/4− cycle shifted magnetoresistance oscillations^3^ induced by low energy photons in the low magnetic field, high filling factor limit. Associated results have produced new interest in the experimental^1–39^ and theoretical^40–62^ study of photo-excited transport in low dimensional systems. The dark properties of the GaAs/AlGaAs 2D material system in the low magnetic field limit have also attracted recent experimental attention as the results have helped to provide new insights into both an observable giant magnetoresistance in the 2DES, and a small, narrow negative magnetoresistance near null magnetic field^63–77^. So far as the latter effect is concerned^74,78,79^, early reports^74^ examined size effects and the phase-breaking rate of good quality GaAs/AlGaAs 2DES material from the 1980’s, while in the lower mobility (≈10^5^ *cm*^2^*V*^−1^*s*^−1^) 2DES^78,80,81^, the temperature dependent conductivity drop at the null magnetic field was viewed as either localization or carrier interaction effect. In the modern era, Bockhorn *et al*. reported a temperature invariant small and narrow magnetoresistance effect around zero magnetic field as a sign for the absence of weak localization^82^ in high quality GaAs/AlGaAs 2DES samples^71,73^. It turns out that the origin of the small and narrow negative magnetoresistance effect about null magnetic field in these ultra high mobility samples is still under debate since the low disorder and long elastic mean free path even in mm-scale high mobility GaAs/AlGaAs specimens suggests the absence of diffusive transport - a necessary condition for the observability of weak localization.

Quantum coherent backscattering originates from the constructive quantum interference of electronic wave-functions which return to the origin after propagating along time-reversed paths in a disordered diffusive medium^78,82–86^. This interference effects leads to enhanced carrier back-scattering and, therefore, to an increase in the resistivity above the classical Drude value. The observation of this interference effect requires that the inelastic length, *ł*_*i*_, exceed the elastic length, *l*_*e*_, i.e., *l*_*i*_ > *l*_*e*_. Since inelastic scattering events, such as electron-electron or electron-phonon interactions, and strong magnetic fields destroy the phase-coherence between time reversed trajectories, the quantum correction to the resistivity/conductivity becomes suppressed by higher temperatures, or finite magnetic fields^82,83,85^. Typically, these interference effects are readily observable at low temperature as sharp magnetoresistance peak about *B* = 0.

Our magnetotransport studies have been characterized by two distinct negative magnetoresistance effects in the high quality GaAs/AlGaAs 2DES in the low magnetic field limit: (a) a negative Giant Magneto-Resistance (GMR) effect over larger magnetic fields (−0.15 ≤ *B*≤ 0.15 *T*) and (b) a small and narrow negative magnetoresistance effect in the immediate vicinity of null magnetic field. The dependence of the negative-GMR on the sample size, DC-bias, temperature, and also the interplay between GMR and the radiation induced magnetoresistance oscillations have been reported^72,76,87^. Here, we present the experimental study of the small and narrow negative magnetoresistance effect about *B* = 0. This effort represents an attempt (a) to determine whether a Hikami *et al*. theory^88^ can succeed to model the small and narrow negative- magnetoresistance effect observed about *B* = 0 in the high mobility GaAs/AlGaAs 2D system, and (b) to extract characteristic parameters such as the inelastic length *ł*_*i*_. Thus, experimentally, we followed the small and narrow negative magnetoresistance effect as a function of the temperature, and the data were then fit using the Hikami *et al*. theory, neglecting spin-orbit interactions^88^. The results indicate that *l*_*i*_ < *l*_*e*_ even at the lowest temperatures, while, typically, it is necessary that *l*_*i*_ ≥ *l*_*e*_ for weak localization. Since, in the high mobility GaAs/AlGaAs 2DES, small angle scattering from remote charged impurities predominates, and such scattering mostly influences the single particle scattering length, *l*_*s*_ = *v*_*F*_*τ*_*s*_, rather than the elastic length *l*_*e*_, we have also extracted *τ*_*s*_ from line-shape fits of Shubnikov-de Haas oscillations^89–91^, and evaluated *l*_*s*_. The results show that *l*_*i*_ > *l*_*s*_, suggesting the possibility of a coherent backscattering effect that arises from small angle scattering due to remote charged impurities in the high mobility GaAs/AlGaAs system.

## Results

**Figure 1:**
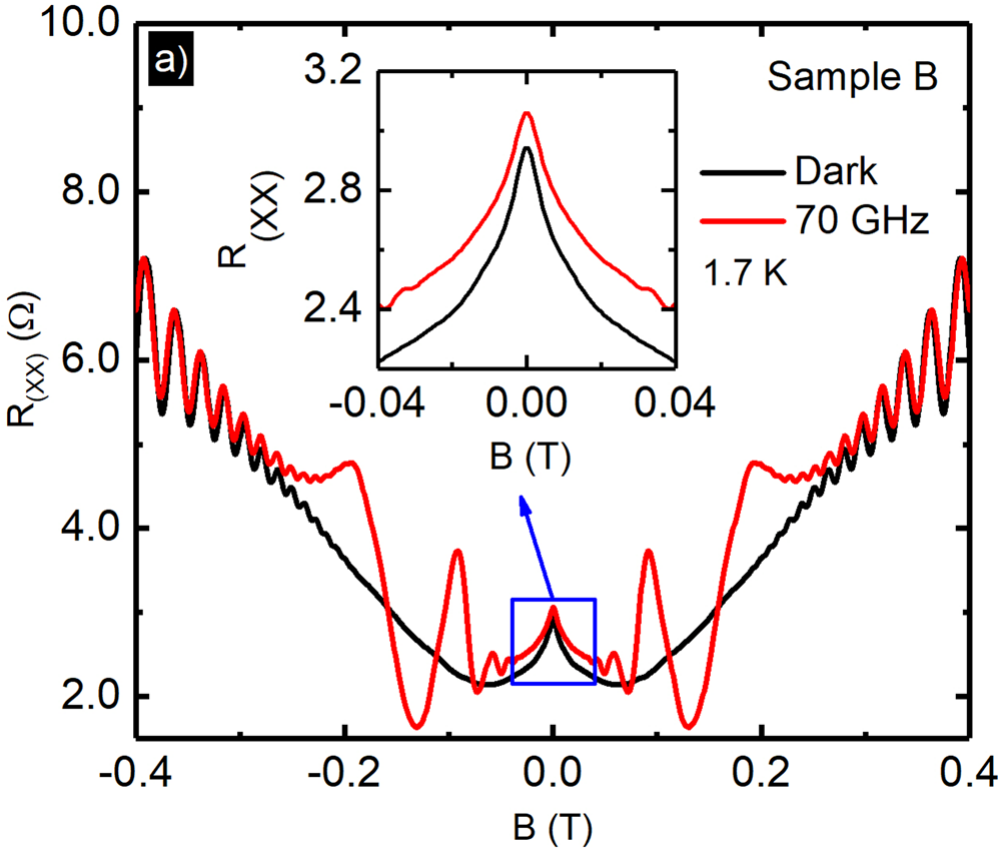
Radiation induced magnetoresistance oscillations in *R*_*xx*_ in a GaAs/AlGaAs heterostructure 2D electron system. *R*_*xx*_ is exhibited vs. the magnetic field, *B*, for dark (Black-curve) and with microwave excitation at *f* = 70 GHz and *P* = 0.77 *mW* (Red-curve). The inset shows the enlarged view of the weak localization-like effect.

Figure 1(a) exhibits magnetoresistance data (*R*_*xx*_) obtained in the dark (black) and under photo-excitation (red) with microwaves at frequency, *f* = 70 *GHz* with a power level *P* = 0.77 *mW*, at *T* = 1.7 *K*. The inset of Fig. 1(a) exhibits an enlarged view of the small and narrow negative magnetoresistance effect, which is the main focus of this study. The small and narrow negative magnetoresistance term spans roughly over −0.025 ≤ *B* ≤ 0.025 *T*, and it is reminiscent of the weak localization effect^72–74,79,82–84,86,92–95^. Note that the shape of the small and narrow negative magnetoresistance term is not significantly affected by the photo excitation, although the *R*_*xx*_ shifts to a higher value under photo-excitation. Figure 1 also indicates that the non-oscillatory portion of the data shows initial negative magnetoresistance to *B* = 0.15 *T*, followed by positive magnetoresistance to *B* = 0.35 *T*, with observable Shubnikov-de Haas oscillations for *B* ≥ 0.2 *T*.^24,27^. The radiation-induced magnetoresistance oscillations, can be observed roughly over the interval −0.2 ≤ *B* ≤ 0.2 *T* (red-curve)^1,3,5–12,14,15,18,20,24,27–30,32–34,36,39^.

**Figure 2:**
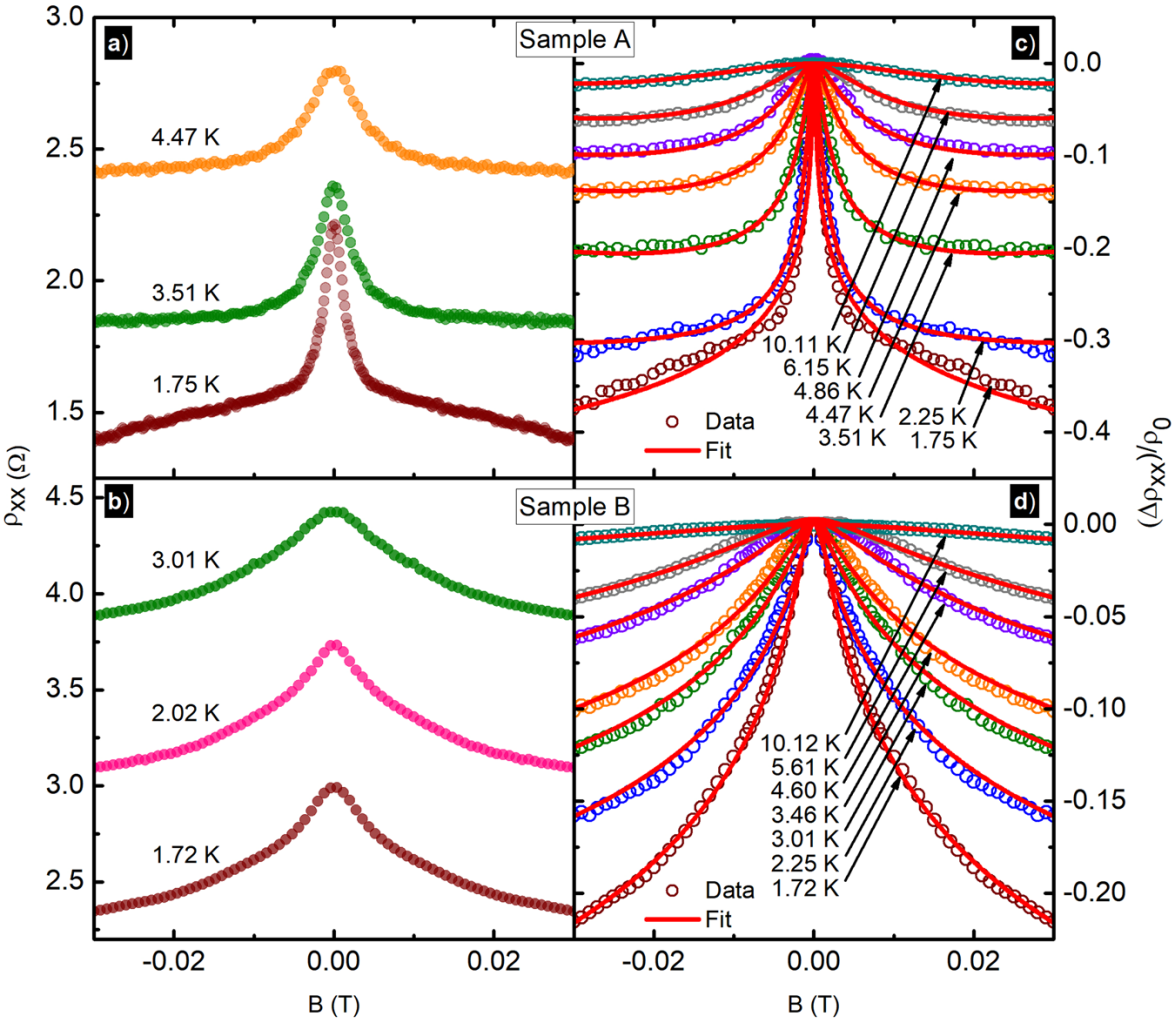
(**a**,**b**) These figures exhibit the magnetoresistivity data (*ρ*_*xx*_) for sample-A and sample-B at selected temperatures, over the span of −0.03*T* ≤ *B* ≤ 0.03*T*. (**c**,**d**) The solid-circles exhibits the normalized experimental data (Δ*ρ*_*xx*_/*ρ*_0_) vs *B* and solid-lines represent the corresponding fits using 2D weak localization theory.

The above-mentioned small and narrow negative magnetoresistance effect is reported here on two samples, labeled sample-A and sample-B, with electron densities *n*_*A*_ = 3.1 × 10^11^ *cm*^−2^, *n*_*B*_ = 2.4 × 10^11^ *cm*^−2^ and electron mobilities *μ*_*A*_ = 1.37 × 10^7^ *cm*^2^/*Vs*, *μ*_*B*_ = 1.13 × 10^7^ *cm*^2^/*Vs*, respectively, over the temperature range 1.7  ≤  *T* ≤ 20.5 *K*. Figure 2(a) and (b) exhibit the raw magnetoresistivity data for the two samples at selected temperatures. The resistivity of the sample increases with increasing temperature as expected and the sample- A shows a much narrower negative magnetoresistance effect compared to the sample-B. In both samples, the FWHM of the peak increases with the increase of the temperature. We examined the small and narrow negative magnetoresistance effect using 2D weak localization theory.

In the absence of spin-orbit scattering, the 2D weak-localization correction to the resistivity is given by,

1 $${\rho }_{(B)}={\rho }_{\mathrm{(0)}}-\frac{{e}^{2}{\rho }^{2}}{2{\pi }^{2}\hslash }[\psi (\frac{1}{2}+\frac{{B}_{i}}{B})+ln\frac{B}{{B}_{i}}]$$

Here, *ψ* is the digamma function, *ρ* resistivity, and

2 $${B}_{i}=\frac{\hslash }{4e{l}_{i}^{2}}$$

We fit the small and narrow negative magnetoresistance effect to equation (1) to extract *l*_*i*_ from the data^74,78,79,84,86,88,92,94,96^. Solid lines in Fig. 2(c) and (d) exhibit fits to the data of sample-A and sample-B respectively at selected temperatures. Note that the data and fit results in the Fig. 2(c) and (d) are shown as (Δ*ρ*_*xx*_/*ρ*_0_) for the sake of clarity. Here, the maximum change in the resistivity (Δ*ρ*_*xx*_/*ρ*_0_) due to the narrow negative-MR effect in sample-A at *T*≈1.7 *K* is (Δ*ρ*_*xx*_/*ρ*_0_) = 0.37 and that of the sample-B is (Δ*ρ*_*xx*_/*ρ*_0_) = 0.22. Thus, the small and narrow magnetoresistance effect is actually rather a substantial effect.

**Figure 3:**
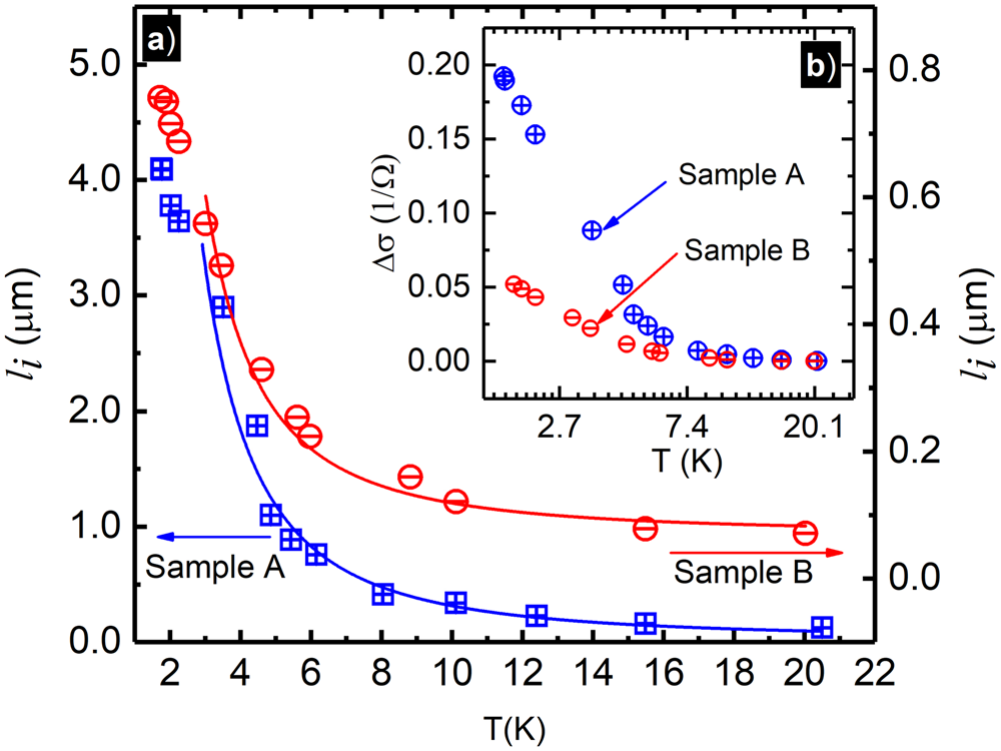
(**a**) This figure exhibits the temperature dependence of the inelastic scattering length *l*_*i*_, for sample-A (Blue) and sample-B (Red), Solid lines correspond to *T*^−2^ law curves. Inset (**b**) shows Δ*σ* vs *Ln*(*T*), Δ*σ* the localization-like correction to the Drude conductivity in 2D, sample-A (Blue) and sample-B (Red).

Figure 3(a) exhibits the temperature dependence of the inelastic length *l*_*i*_ for the sample-A (Blue) and sample-B (Red), calculated using the fit extracted parameter *B*_*i*_. The *l*_*i*_ at the base temperature *T* = 1.7 *K* of the samples A and B are 4.10 *μm* and 0.76 *μm*, respectively, and *l*_*i*_ decreases monotonically with increasing temperature. In comparison, the elastic scattering length *l*_*e*_ for the samples-A and B are 123 *μm* and 79 *μm* respectively, and the characteristic width of the Hall bar sample is *W* = 200 *μm*. Thus, the order of magnitude of *W* and *l*_*e*_ are the same although *W* > *l*_*e*_. On the other hand, the *l*_*i*_≪*l*_*e*_, roughly by a factor of 100. Sample-A with relatively higher electron density and mobility exhibits greater *l*_*i*_ and it differs from the sample-B by a factor of ≈5. The fit extracted *l*_*i*_ values of both samples follows *T*^−2^ law curves above ≈3 *K*, and data deviates from the curves showing a tendency of saturation at lower temperatures. The *T*^−2^ behavior of the *l*_*i*_ in the given temperature range suggests that the inelastic scatterings of these samples may be mainly electron-electron type^74,78,81,97^. Figure 3(b) exhibits the conductivity change, Δ*σ* as a function of the temperature for the two samples. Sample-A shows greater change in the conductivity for the small and narrow negative magnetoresistance effect. In both specimens, the small and narrow magnetoresistance effect becomes vanishingly small above ≈10*K*. Note that the logarithmic temperature dependence of the conductivity correction in the low-temperature limit can be viewed as a signature of weak localization- effect in the 2DES^98,99^.

**Figure 4:**
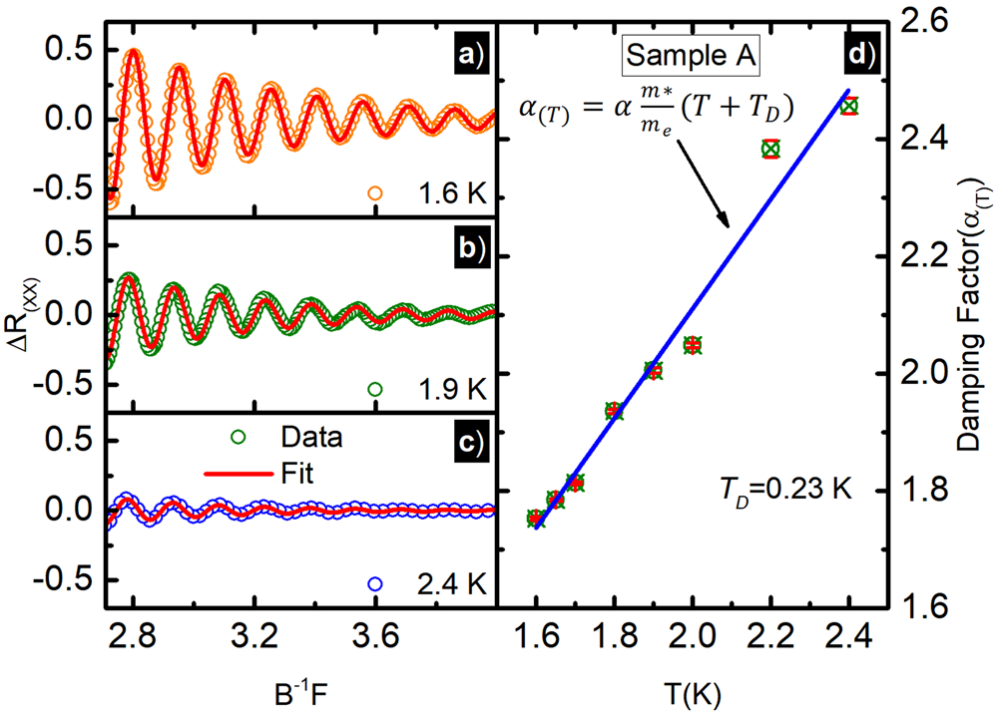
(**a**–**c**) This figures exhibit the background subtracted SdH-Oscillations, i.e. Δ*R*_*xx*_ vs *B*^−1^*F* (open circles) and numerical fit (red-solid lines) to equation (3), i.e. ${\rm{\Delta }}{R}_{xx}^{fit}=A\,exp(-\,{\alpha }_{(T)}/B)cos\mathrm{(2}\pi F/B)$, at different temperatures, *F* is the oscillations frequency and *α*_(*T*)_ = α m^*^/*m*_*e*_(*T* + *T*_*D*_) (see the text). (**d**) Exhibits the fit extracted temperature dependent damping factor *α*_(*T*)_ vs *T* for the sample-A.

As noted above, measurements suggest that *l*_*i*_ < *l*_*e*_ in these quasi ballistic samples, which seems, at the outset, to rule out weak localization. However, it is known that small angle scattering from remote ionized impurities is predominant in the high mobility GaAs/AlGaAs 2D system and, as a consequence, the *τ*_*e*_ can exceed the single particle lifetime, *τ*_*s*_, by factor of 100. (The *τ*_*e*_ reflects mostly large angle scattering while *τ*_*s*_ reflects all scattering, including small angle scattering.) Thus, we utilized lineshape fits to determine the single particle lifetime *τ*_*s*_, and thereby the single particle length, *l*_*s*_=*v*_*F*_*τ*_*s*_, which reflects all scattering events, from the low-field SdH oscillations^89–91^. Fig. 4(a–c) (open-circles) shows the SdH oscillations vs *B*^−1^, scaled with oscillation frequency *F* for sample-A at some selected temperatures. Figure 4(a–c) red-solid lines exhibit the fit using equation (3).

3 $${\rm{\Delta }}{R}_{xx}^{fit}=A\,exp(-\,{\alpha }_{(T)}/B)cos\mathrm{(2}\pi F/B)$$

Here, *α*_(*T*)_ = *α* (m*/*m*_*e*_)(*T* + *T*_*D*_), *α* = 14.69 *T*/*K*, m*/*m*_*e*_ = 0.064 for these samples and *T*_*D*_ is the Dingle temperature. The intercept of the graph of *α*(*T*) vs *T* (i.e. Fig. 4(d)), determines the value of *T*_*D*_. The *τ*_*s*_ is related to *T*_*D*_ through the expression *τ*_*s*_ = ℏ/2*πk*_*B*_*T*_*D*_, where *k*_*B*_ is the Boltzmann constant. As shown in the Fig. 4(d), the calculated Dingle temperature for the sample-A is *T*_*D*_ = 0.23±0.15*K* and the corresponding *τ*_*s*_ for the sample-A is *τ*_*s*_ = 4.86 × 10^−12^ *s*. Thus, for sample A, *l*_*s*_ = *v*_*F*_*τ*_*s*_ = 1.26 *μm*, and *l*_*i*_ > *l*_*s*_. This result suggests the possibility that the observed small and narrow negative magnetoresistance effect can be understood as coherent backscattering if small angle scattering due to remote ionized impurities is responsible for the scattering-induced closed electronic trajectories involved in weak localization.

## Discussion

Magnetotransport studies of the ultra high mobility GaAs/AlGaAs 2DES exhibit a small and narrow negative magnetoresistance effect that appears around zero field^72,73,76,87^. This work aimed to follow the effect of temperature on the observed small and narrow negative magnetoresistance effect, determine whether a weak localization type line-shape analysis succeeds in describing the data, extract physical parameters, and possibly identify the physical origin of the observed effect. Thus, systematic measurements of the *R*_*xx*_ were carried out as a function of temperature ranging from 1.7 *K* to 20.5 *K* for two different samples and the data were fit using the Hikami 2D WL theory^88^, neglecting the spin orbit scattering term, and also electron-electron interaction effects. The neglect of elecctron-electron interactions effects can be justified by the absence of an observable concurrent correction to the Hall coefficient^72^.

With the increase of the temperature, the FWHM of the small and narrow negative-magnetoresistance effect increases and the peak height decreases, see Fig. 2(a,b), while it is restricted to weak magnetic fields around *B* = 0 *T*, see Fig. 1. This narrow peak is similar in appearance to the typical temperature dependent weak localization phenomena^78,82,85,92,100^. In canonical weak localization, the magnetoresistance effect disappears with increasing temperature as *l*_*i*_, which initially exceeds *l*_*e*_ at the lowest temperatures, becomes smaller and, eventually, comparable with the elastic scattering length *l*_*e*_, i.e., *l*_*i*_ ≈ *l*_*e*_. In both samples, the fit extracted inelastic length, *l*_*i*_, see Fig. 3(a), monotonically decreases with increasing temperature. For the data shown here, in addition to the reduction in the inelastic scattering length with increased *T*, a result obtained through fits, quenching of the narrow negative-MR effect at higher temperatures is also confirmed by the reduced conductivity change *δσ* with increasing *T*, see the inset of Fig. 3(b). A relatively stronger small and narrow negative-magnetoresistance effect is observed here in the sample A with the higher mobility/density. Figure 3(a) also indicates that the *l*_*i*_ in sample-A exceeds the *l*_*i*_ in sample-B throughout the examined temperature range. It is expected that a sample with higher mobility/density will exhibit a relatively larger *l*_*i*_ than a lower mobility/density sample^83^.

The elastic scattering length *l*_*e*_ for the samples-A and B are 123 *μm* and 79 *μm* respectively, and the characteristic width of the Hall bar sample is *W* = 200 *μm*, i.e., the order of magnitude of *W* and *l*_*e*_ are the same although *W* > *l*_*e*_. Thus, these specimens satisfy the quasi ballistic transport condition, while weak localization is a characteristic of diffusive transport. In these device structures, a high mobility of about 10^7^*cm*^2^/*Vs* is achieved by utilizing the remote *δ*-doping technique. In this technique, scatterings due to ionized impurities are minimized while maintaining a higher electron concentration by separating the ionized impurities associated with donors from the 2D-electron layer^101,102^. In these high mobility specimens where scattering is predominantly due to long range Coulomb potentials from remote charged impurities, most scattering is expected to be of the small-angle variety. Since such scattering does not impact so much the transport time, one expects the elastic length, *l*_*e*_, which is of the same order as the sample width, to not be the parameter of interest for comparison, as far as quantum coherent backscattering is concerned in this 2DES. Thus, we measured the single particle lifetime, *τ*_*s*_, which tends to count all scattering, including small angle scattering, events. Figure 4 exhibits the evaluation of the temperature damping the SdH-oscillations that is used to calculate the *τ*_*s*_ and determine *l*_*s*_ = *v*_*F*_*τ*_*s*_. Note that the ratio *l*_*e*_/*l*_*s*_ ≈ 100 in these specimens. This indicates that remote ionized impurity scattering, which favors small angle scattering, is predominant and significant in our samples, as reported by others for other modulation-doped GaAs/AlGaAs 2DES^89–91^. Therefore, we have reasoned that the criterion for the observability of coherent backscattering in such specimens is that *l*_*i*_ > *l*_*s*_ holds true, rather than *l*_*i*_ > *l*_*e*_, as is usual for weak localization. Indeed, it turns out that while *l*_*i*_ > *l*_*e*_ is not satisfied in these specimens, the proposed alternate condition *l*_*i*_ > *l*_*s*_ holds true. This feature has suggested the interpretation that the observed small and narrow negative magnetoresistance effect observed about zero magnetic field is coherent backscattering induced by small angle scattering from remote ionized impurities in the high mobility GaAs/AlGaAs 2DES.

A different explanation for this small and narrow negative-magnetoresistance effect around *B* = 0 in GaAs/AlGaAs 2DES has been given by Bockhorn *et*. *al*.^71,73^. Their measurements, carried out below *T* = 800 *mK*, exhibited a similar sharp negative-magnetoresistance effect around zero field that turned out, however, to be temperature independent. They suggested that the small and narrow negative magnetoresistance effect is induced both by rare strong scatters due to the presence of macroscopic defects and remote ionized impurities. In this regard, note that magnetoresistance saturation due to a saturation of the inelastic length at low temperatures, as *T* → 0, is known^65,83^.

## Conclusions

In summary, this study examines a small and narrow-negative magneto resistance effect that appears around *B* = 0 *T* in the high mobility, *μ* ≈ 10^7^*cm*^2^/*Vs*, GaAs/AlGaAs 2DES. This work reports the influence of sample temperature (1.7 ≤ *T* ≤ 20 *K*) and carrier density/mobility on the *l*_*i*_, that are extracted using a Hikami *et al*. line-shape analysis^88^. The fit determined *l*_*i*_ decreases with increasing temperature, per expectations. The results indicate that *l*_*i*_ < *l*_*e*_ at all temperatures, which is not what would be expected for canonical weak localization. However, since these specimens are characterized by charged impurity scattering from remote donors, scattering is expected to be predominantly of the small angle variety. Thus, we measured *τ*_*s*_ and *l*_*s*_, the single particle lifetime and mean free paths, respectively, which take into account the small angle scattering. It turns out that *l*_*i*_ > *l*_*s*_, which suggests the interpretation that the observed small and narrow negative magnetoresistance effect originates from coherent backscattering due to small angle scattering from remote ionized donors in the high mobility GaAs/AlGaAs 2DES.

## Methods

High mobility MBE grown GaAs/AlGaAs heterostructures that consist of 5000 Å GaAs-substrate/100 × (100 Å AlGaAs | 30 Å GaAs)-Superlattice/12000 Å GaAs/700 Å AlGaAs/5 Å Si *δ*-doping/2400 Å AlGaAs/100 Å GaAs layers, were patterned into Hall bars by photolithography. The doping of the Si *δ*-layer is about ≈10^12^ *cm*^−2^. Four terminal electrical measurements were carried out on the Hall bars using low-frequency lock-in based techniques with the sample mounted at the end of a cylindrical waveguide, within a variable temperature insert, inside a superconducting solenoid in the *B* ⊥ *I* configuration. Since the 200 *μm* wide Hall bars included voltage probes spaced by 200 *μm*, the effective Length-to-Width (L/W) ratio for the measurements presented here is L/W = 1. The samples were immersed in liquid helium, and temperature control was realized by controlling the vapor pressure of liquid helium. Typically, magnetic field (*B*) sweeps of the lock-in detected diagonal voltage *V*_*xx*_ were collected at a fixed temperature, *T*, in order to determine *R*_*xx*_ = *V*_*xx*_/*I*_*ac*_.
